# The effects of a nurse-led integrative medicine-based structured education program on self-management behaviors among individuals with newly diagnosed type 2 diabetes: a randomized controlled trial

**DOI:** 10.1186/s12912-022-00970-7

**Published:** 2022-08-05

**Authors:** Xingfeng Yu, Janita Pak Chun Chau, Lanting Huo, Xiaomei Li, Dan Wang, Hongjuan Wu, Yulian Zhang

**Affiliations:** 1 The Nursing Department, Shaanxi Provincial People’s Hospital, Xi’an, Shaanxi 710068 People’s Republic of China; 2 The Nethersole School of Nursing, The Chinese University of Hong Kong, Shatin, New Territory, Hong Kong; 3 Faculty of Nursing, Health Science Center, Xi’an Jiaotong University, Xi’an, Shaanxi 710061 People’s Republic of China; 4 School of Public Health, Health Science Center, Xi’an Jiaotong University, Xi’an, Shaanxi 710061 People’s Republic of China; 5 The Director’s Office, Shaanxi Provincial People’s Hospital, Xi’an, Shaanxi 710068 People’s Republic of China

**Keywords:** Type 2 diabetes, Structured education, Culture-tailored interventions, Self-management, Glycemic control, Self-efficacy

## Abstract

**Background:**

International guidelines advocate providing prompt structured education to individuals with diabetes at diagnosis. However, among the few eligible structured education programs, heterogeneous intervention regimens and inconsistent findings were reported. Eligible programs for Chinese individuals with diabetes are lacking. This study aimed to investigate the effects of a nurse-led integrative medicine-based structured education program on self-management behaviors, glycemic control and self-efficacy among individuals with newly diagnosed type 2 diabetes.

**Methods:**

Employing a randomized controlled trial, 128 individuals with type 2 diabetes diagnosed in the preceding three to nine months were recruited from four university-affiliated tertiary hospitals in Xi’an City, Northwest China, and randomly allocated to the intervention or control groups after baseline assessments. Participants in the intervention group received a 4-week nurse-led integrative medicine-based structured education program, which is theoretically based on the Health Belief Model and Self-Efficacy Theory, in line with updated diabetes management guidelines, and informed by relevant systematic reviews. Participants in the control group received routine care. Self-management behaviors and self-efficacy were measured with the Summary of Diabetes Self-Care Activities and the Diabetes Management Self-Efficacy Scale at baseline, immediate post-intervention and 12 weeks following the intervention while Glycated Hemoglobin A was measured at baseline and the 12th-week follow-up. The intervention effects were estimated using the generalized estimating equation models.

**Results:**

Participants in the intervention group exhibited significantly better self-management performance in specific diet regarding intake of fruits and vegetables at both follow-ups (β = 1.02, *p* = 0.011 and β = 0.98, *p* = 0.016, respectively), specific diet regarding intake of high-fat foods at the immediate post-intervention follow-up (β = 0.83, *p* = 0.023), blood glucose monitoring at the 12th-week follow-up (β = 0.64, *p* = 0.004), foot care at both follow-ups (β = 1.80, *p* <  0.001 and β = 2.02, p <  0.001, respectively), and medication management at both follow-ups (β = 0.83, *p* = 0.005 and β = 0.95, *p* = 0.003, respectively). The intervention also introduced significant improvements in Glycated Hemoglobin A (β = − 0.32%, *p* <  0.001), and self-efficacy at both follow-ups (β = 8.73, p <  0.001 and β = 9.71, p <  0.001, respectively).

**Conclusions:**

The nurse-led integrative medicine-based structured education program could produce beneficial effects on multiple diabetes self-management behaviors, glycemic control and self-efficacy.

**Trial registration:**

This study was retrospectively registered in the *ClinicalTrials.gov**.* on 25/08/2017; registration number: NCT03261895.

**Supplementary Information:**

The online version contains supplementary material available at 10.1186/s12912-022-00970-7.

## Background

The alarming prevalence and incidence of diabetes and its multidimensional negative consequences have introduced tremendous burdens to individuals with the condition, their families, and the healthcare system worldwide [1, 2]. Optimal glycemic control is the key to prevent the progression of diabetes and the development of diabetes-related complications. However, despite the strong advocacy of reasonable individualized glycemic targets [2, 3], the majority of individuals with diabetes failed to reach such targets [4]. Due to the chronic nature of diabetes, individuals with the condition spend most of their time in the community, rather than hospitals; the individuals and their caregivers carry the major responsibilities regarding their daily disease management [5]. As such, individuals with diabetes need to acquire sufficient knowledge and skills about, and positive attitudes towards diabetes self-management. Unfortunately, ample researches have shown that individuals with diabetes, especially those with newly diagnosed diabetes, had suboptimal self-management knowledge and competence [6].

Diabetes education, equivalently referred to as diabetes self-management education and support (DSMES), is an effective and cost-efficient approach for individuals with diabetes to initiate self-management endeavors at the first diagnosis and to maintain effective self-management behaviors throughout their lifetime [7]. Research evidence has frequently favored the benefits of DSMES in improving knowledge, motivation, and skills in diabetes self-management, which further lead to improved biomedical, psychosocial and behavioral outcomes [8]. The National Institute for Health and Care Excellence (NICE) suggests providing structured education to individuals with diabetes once the diagnosis is made [9]. First defined by NICE, structured education refers to a planned and graded program that is comprehensive in scope, flexible in content, responsive to an individual’s clinical and psychological needs and adaptable to his/her educational and cultural background [10]. The key criteria of a structured education program include 1) a clear underlying philosophy on which the program is based; 2) a structured written curriculum; 3) trained health educators who are familiar with the program and its delivery; 4) a quality assurance system for the structure, process, contents and delivery of the program; and 5) an audit of the program outcomes, including the biomedical and psychosocial outcomes [11, 12]. Even though not named as structured education, the American Diabetes Association (ADA) and the Association of Diabetes Care & Education Specialists (ADCES, formerly known as the American Association of Diabetes Educators) jointly proposed 10 equivalent criteria in the national standards for DSMES [7].

Our research team conducted a systematic review of randomized controlled trials to investigate the effectiveness of structured education programs for individuals with type 2 diabetes [13]. The extensive search strategy only identified seven eligible trials, and due to the obvious and substantial heterogeneity in the intervention regimen of these trials, the attempt to quantitatively synthesize the results with meta-analysis failed. Narrative summaries of the results demonstrated inconclusive effects of structured education programs on glycemic control, self-efficacy, self-management behaviors and health-related quality of life among individuals with type 2 diabetes [14–21]. Considering the paucity of eligible structured education programs and the variety in the method of intervention delivery, content, intervener, use of technology and underpinning philosophy of existed trials, further interventions tailored to specific populations are desirable to determine the effectiveness of structured education programs for individuals with type 2 diabetes [8].

Culture-tailored interventions have frequently been advocated by healthcare professionals and researchers. However, to date, publications on eligible structured education programs for Chinese individuals with type 2 diabetes are vacant. As China has the largest diabetes population [1], it is of great significance to develop and evaluate the effectiveness of culturally appropriate structured education programs in Chinese individuals with type 2 diabetes. Traditional Chinese medicine (TCM), an important branch of alternative medicine that deeply rooted in the traditional Chinese culture, proposed a variety of lifestyle modifying interventions for individuals with diabetes, such as dietary intervention based on the TCM body constitution theory, exercises including tai chi and ba duan jin (eight-session brocade qigong), and TCM-based psychological care. The effectiveness of such interventions for individuals with type 2 diabetes has been demonstrated in a recent systematic review with meta-analysis [22]. Integrative medicine, which combines conventional diabetes management with alternative interventions, has gained popularity in type 2 diabetes treatment worldwide.

Lifestyle modification programs should be theory-driven as this approach helps to identify what is relevant, important and feasible to produce the anticipated outcomes, and thus to inform the contents and delivery of the interventions [23]. In this study, the Health Belief Model and Self-Efficacy Theory were used as the theoretical ground. The development of the interventions was also informed by the most updated diabetes management guidelines and relevant systematic reviews. This study was featured with the incorporation of traditional Chinese medicine-based lifestyle interventions in a structured diabetes education program. The objectives of this randomized controlled trial were to evaluate the effects of a nurse-led integrative medicine-based structured education program on diabetes self-management behaviors, glycemic control and self-efficacy among individuals with newly diagnosed type 2 diabetes. The alternative hypothesis was as follows: compared to the counterparts in the usual care control group, participants who received the structured education program would have significantly higher level of diabetes self-management behaviors, glycemic control (indicated by Glycated Hemoglobin A, HbA1c) and self-efficacy.

## Methods

The reporting of this study adhered to the Consolidated Standards of Reporting Trials (CONSORT) 2010 statement [24].

### Study design and settings

This was a prospective, outcome assessor-blinded, randomized controlled trial with a 1:1 parallel-group design (registration identifier: NCT03261895) conducted at four university-affiliated, tertiary hospitals in Xi’an City, Northwest China, from May 2017 to December 2017. All participating institutions are governmental referral hospitals which are homogeneous with regard to size, grade of certification, diabetes treatment routine and laboratory equipments.

### Participants

The inclusion criteria for the study participants were as follows: 1) community dwellers with sufficient ability to commute between their community and the research sites; 2) diagnosed with type 2 diabetes by a physician and received anti-hyperglycemic treatments in the inpatient wards of the four hospitals in the preceding three to nine months (individuals diagnosed within three months were not included considering a remarkable decrease in glycemic level is frequently observed in the period after the diagnosis due to intensive medical therapy [12], which may compromise the effects of the study intervention); 3) aged ≥18 years; 4) taking medications (including insulin) or not; and 5) consent to participate in the study. The exclusion criteria were as follows: 1) with a clinical diagnosis of mental disorders; 2) with a terminal illness; 3) physically disabled; 4) taking part in other studies; and 5) with aural or visual problems.

### Sample size estimation

The equation for the sample size estimation of a statistical superiority randomized controlled trial comparing two parallel-sample means: $N=2{\left(\frac{t_{1-\alpha /2}+{t}_{1-\beta }}{\delta /\sigma}\right)}^2$ was employed to determine the sample size. Four parameters are required to estimate the sample size: type I error (α), type II error (β), the expected intervention effect (δ) and its standard deviation (σ) in the population for a given outcome variable (usually the primary outcome). As was demonstrated in a systematic review, group-based diabetes education can further improve self-management by a standardized mean difference of 0.55 (δ/σ) compared with usual care among individuals with type 2 diabetes [25]. Thus, 64 participants for each group were necessary to detect a between-group difference at a 5% significance level with 80% power (1- β), allowing for a drop-out rate of 15%.

### Sampling

This study employed a two-level sampling approach. The first-level was conducted at the research site level on a convenient basis, while the second-level was carried out at the individual level within the research sites using simple random sampling. One trained diabetes education specialist who was responsible for the routine telephone follow-ups in the Department of Endocrinology at each hospital assessed the eligibility of potential participants. After identifying a potential participant, the nurse briefly introduced the program to the participant and invite he/she to join. The nurses recorded the information of interested participants and thus, a sampling pool for each hospital was available. Two trained research assistants who were not involved in other procedures of the study sequentially coded the interested participants based on the time when their information was recorded and then generated a set of random numbers for each hospital to sample the research participants using Research Randomizer version 4.0.

### Random allocation

Eligible participants were randomly allocated to receive either the nurse-led integrative medicine-based structured education program or usual care at a 1:1 ratio employing simple randomization with Research Randomizer version 4.0. Random numbers with pre-specified group code were kept in uniform, opaque and sealed envelopes. After getting written consent from eligible participants, baseline assessments were conducted. Then, the enrolling investigators asked the participants to pick an envelope to decide their group assignment. Concealed allocation was maintained as the trained research assistants who prepared the envelopes and the enrolling investigators were not involved in other procedures of the study. Meanwhile, the trained outcome assessors were blinded to the group allocation throughout the study period.

### Interventions

Participants in both groups received the standard diabetes management provided by the community healthcare centers, which, according to the governmental policy, provide primary healthcare services to all the citizens within their regions. The standard community diabetes management includes building health records, providing primary medical treatments and health education, arranging regular health check-ups and home visits, etc. In addition to the standard community diabetes management, participants in the intervention group received the nurse-led integrative medicine-based structured education program while those in the control group received the control intervention.

#### Nurse-led integrative medicine-based structured education program

Based on a theoretical framework of the Health Belief Model and Self-Efficacy Theory (Supplementary Fig.1) [26–28], this nurse-led integrative medicine-based structured education program was developed in accordance with updated evidence-based international and national diabetes management guidelines [2, 3, 7, 9, 29] and the findings of systematic reviews regarding the effectiveness of structured education and TCM-based lifestyle interventions for individuals with type 2 diabetes [13, 22]. The theoretical frameworks involved six constructs, and in the intervention sessions, various strategies were employed to improve the participants’ perceived susceptibility to uncontrolled diabetes and complications, perceived seriousness of uncontrolled diabetes and complications, perceived benefits of taking self-management behaviors, cues to action, and self-efficacy (derives from for sources: performance accomplishments, vicarious experience, verbal persuasion and emotional arousal), and to decrease the participants’ perceived barriers to taking self-management behaviors (Detailed in Table 1).

**Table 1:** Details of the intervention and strategies to address the constructs in the theoretical frameworks

Scheduled activities	Description of intervention and strategies to address the constructs in the theoretical frameworks
**Baseline assessment and goal setting**	• Identify the participants’ Traditional Chinese Medicine syndromes; • Measure the participants’ health outcomes; let the participants know the results and tell them the implications *(Cues to action)*; • Encourage the participants to set realistic goals and the goal-obtaining evaluation intervals for themselves *(Cues to action; Verbal persuasion; Performance accomplishments)*; • Remind the participants of the potential consequences of setting a too easy or too hard goal *(Cues to action; Verbal persuasion)*.
**Topic-based structured education**	
Topic 1: Basic knowledge about diabetes	• Teach the participants about the basic knowledge of the disease, including the physiological process of normal glucose metabolism and the abnormal process that lead to the disease *(Perceived susceptibility; Perceived severity; Cues to action)*; • Discuss the consequences, various complications, and cost of the disease and the fact that these consequences can be prevented or delayed with proper treatment and management *(Perceived susceptibility; Perceived severity; Cues to action)*; • Introduce the treatments of diabetes; highlight the importance and core aspects of lifestyle modification *(Perceived benefits of taking action; Cues to action; Verbal persuasion)*.
Topic 2: Proper physical activities	• Address the importance of regular physical activity for diabetic control *(Cues to action; Verbal persuasion)*; • Identify common misconceptions regarding physical activity *(Perceived barriers to taking action; Cues to action)*; • Let the participants know the core principles of doing exercise, for example, necessary warming-up and cooling-down, sufficient duration and intensity *(Cues to action)*; • Introduce the benefits and advantages of ba duan jin, instruct them to practice ba duan jin step-by-step based on the video and printed materials published by the General Administration of Sport of China, and arrange reinforcement sessions to provide correction and/or further instructions following the educational sessions in week 2–4 (reinforcement sessions lasted for around 15 minutes) *(Cues to action; Performance accomplishments)*; • Encourage the participants to practice ba duan jin for at least 150 minutes and at least 5 days each week *(Cues to action; Verbal persuasion)*.
Topic 3: Healthy dietary behaviors	• Address the importance of healthy eating behaviors *(Cues to action; Verbal persuasion)*; • Identify common misconceptions regarding dietary behaviors *(Perceived barriers to taking action)*; • Teach the participants about the principles of healthy eating *(Cues to action; Verbal persuasion)*; • Let the participants know the importance of dietary constraints including sugar and salt intake; Teach them to identify and avoid foods that are sugar-rich, salt-rich or oil-rich. Use assistive tools to help demonstrate the messages; For example, when suggesting the participants take less than 6 g salt per day, use weight spoons or beer bottle caps to help the participants to have a better concept of how much salt weights 6 g *(Cues to action; Verbal persuasion)*; • Give individualized food recommendations based on the participants’ Traditional Chinese Medicine syndromes *(Cues to action; Verbal persuasion).*
Topic 4: Regular monitoring and diabetes complications surveillance	• Address the importance of regular self-monitoring of blood glucose *(Cues to action; Verbal persuasion)*; • Identify barriers to do regular self-monitoring *(Perceived barriers to taking action)*; • Teach the participants skills in conducting self-monitoring of blood glucose through various methods, for example, demonstration and back-demonstration, and how to interpret the results *(Cues to action; Performance accomplishments)*; • Propose regular checks of other related health indicators, including HbA1c, blood pressure, lipids, and body weight (such of regular follow-up plan is rarely addressed by the healthcare professionals in Mainland China) *(Cues to action; Verbal persuasion)*; • Introduce the common diabetes complications and their manifestations, such as hypertension, foot ulcers and diabetic ketoacidosis; address the importance of diabetes complications surveillance and early treatments once diabetes complications are detected *(Cues to action; Verbal persuasion)*.
Topic 5: Taking medication	• Address the importance of adhering to continuous medication therapy and medication adherence *(Cues to action; Verbal persuasion)*; • Introduce the commonly used oral medicines and insulins, let the participants know the potential adverse drug effects of medicines, for example, hypoglycemia *(Cues to action)*; • Teach the participants how to store, prepare and inject insulin by demonstration and back-demonstration; Remind the participants to seek for professional help when their blood glucose control is suboptimal or the presence of adverse drug effects *(Perceived barriers to taking action; Cues to action; Performance accomplishments; Verbal persuasion)*.
Topic 6: Risk factors management	• Discuss the potential risk factors individuals with diabetes may encounter, such as smoking, alcohol abuse, and excessive exercise *(Perceived barriers to taking action)*; • Discuss the strategies to avoid the risk factors and the strategies to address the risk factors one presented *(Cues to action; Verbal persuasion)*.
Topic 7: Problem solving	• Discuss the common problems individuals with diabetes may encounter, for example, managing diabetes during a business trip or during a journey *(Perceived barriers to taking action)*; • Discuss the possible solutions to address the common problems *(Cues to action; Verbal persuasion)*; • Introduce the available formal or informal institutions where participants can seek for help or social supports, such as the community health service centers and the support groups organized by the diabetic peers *(Cues to action)*.
Topic 8: Healthy coping	• Address the importance of a healthy attitude toward living with diabetes *(Cues to action; Verbal persuasion)*; • Discuss the common psychological problems that may happen to individuals with diabetes *(Perceived barriers to taking action)*; • Introduce various strategies to avoid the situations that trigger these psychological problems or relieve them once presented (*Cues to action; Emotional arousal*); • Remind the participants to seek for professional help once the problems are serious or last for a long time *(Cues to action; Verbal persuasion)*; • Introduce the real-life examples and invite peers to show the participants how their counterparts gained success over the management of the conditions *(Vicarious experience)*; • Encourage participants that they can also gain good diabetic control through proper self-management behaviors and that even the most difficult problems can be resolved or managed through persistent endeavors *(Verbal persuasion)*.

The study intervention was a group-based structured education program with a group size of 16, informed by the recommendation of Centers for Medicare and Medicaid Services that a desired group size for patient education of two to 20 [30] and the consideration of cost-efficiency. Upon commencement of the intervention, the research participants were provided with the measurement results of their health indicators and encouraged on realistic goal setting. The 4-week program with eight interactive educational sessions was delivered by trained clinical nurses at the participating hospitals following the intervention protocols. Each educational session focused on one of the comprehensive diabetes management topics, including basic knowledge about diabetes, proper physical activities, healthy dietary behaviors, taking medication, regular monitoring and diabetes complications surveillance, risk factors management, problem solving, and healthy coping. Details of the intervention are presented in Table 1. A printed diabetes knowledge and management handbook in line with the contents of the educational sessions was developed by the research team and handed out to the participants to facilitate the intervention delivery. The handbook was evaluated for its accuracy, adequacy, relevancy and cultural competence for individuals with newly diagnosed type 2 diabetes by an external expert panel.

The TCM components were incorporated in the topics related to healthy dietary behaviors and proper physical activities. Upon group allocation, participants in the intervention group were arranged with a clinic visit with a TCM practitioner, during which the individualized TCM syndrome of each participant was identified following the guidelines of the National Administration of Traditional Chinese Medicine [29]. In the educational session regarding healthy dietary behaviors, apart from the principles of diet management proposed in the modern medical paradigm, the research participants were instructed about individualized foodstuff priority based on their TCM syndromes [29]. In addition to the general physical activity principles, the research participants were instructed to take ba duan jin as a concrete form of physical activity. Ba duan jin, or eight session brocade, is a special type of Tai Chi that combines physical activity with controlled breath and meditation, and has been demonstrated to be beneficial for individuals with type 2 diabetes in a recent systematic review with meta-analysis [22]. The ba duan jin performance was taught to the research participants step-by-step following the printed and video guidelines of the General Administration of Sport of China [31].

Four registered nurses from the research sites were selected as the educators based on the following criteria: (1) had a bachelor degree or above; (2) cared for individuals with diabetes for at least five years; and (3) was a member of the health education team in their department. During a two-day workshop, the principal investigator trained the educators on the objectives and underlying philosophy of the program while a senior nurse who had rich experience in evidence-based nursing practice trained the educators on the contents, delivery skills and procedure of the program. Back-demonstrations were required to guarantee the competence of the educators. The instruction of ba duan jin performance was given by the same Tai Chi master from the Center of Sports of a renowned local university. A validated diabetes knowledge and management handbook was developed to facilitate the delivery of the educational sessions. The intervention fidelity was maintained by utilizing a uniform intervention package (including the study protocol, a set of PowerPoint slides for the program delivery, and the diabetes knowledge and management handbook) by trained interveners and maintaining process audit by the principal investigator and the senior trainer throughout the intervention period.

#### Control intervention

Participants in the control group received the routine diabetes care provided by the participating hospitals, which included brief discharge instructions focused on medicine regimen, general principles of having foods and taking physical activities, suggested plan of follow-up visits, and routine telephone follow-ups. Interested participants in the control group were provided with the validated diabetes knowledge and management handbook at the end of the study.

### Measurement

#### Socio-demographic and clinical profile

Before randomization, a self-designed data collection form was used to collect the socio-demographic and clinical data.

#### Outcome variables

The primary outcome was diabetes self-management behaviors, while the secondary outcomes were HbA1c and self-efficacy. HbA1c was measured at baseline (T0) and 12th-week post-intervention (T2), while other outcomes were measured at baseline (T0), immediate post-intervention (T1) and 12th-week post-intervention (T2). A 12th-week post-intervention measurement is particularly valuable for evaluating the effectiveness of a diabetes education program. For one thing, it enables the assessment of the sustainability of intervention effects. For another, as the golden indicator of glycemic control, HbA1c represents the average glycemic level over an approximate 12-week interval.

Diabetes self-management behaviors were measured with the Summary of Diabetes Self-Care Activities (SDSCA) [5]. SDSCA had 11 core items which assess the informants’ diabetes-related behaviors in the preceding seven days regarding general diet (two items), specific diet (two items: one on the intake of fruits and vegetables while the other on the intake of high-fat foods), physical activity (two items), blood glucose monitoring (two items), foot care (two items) and smoking status (one item). The SDSCA provides 14 additional items that could be used selectively by researchers to meet their research interests. In the current study, the additional item on medication management was included considering that medication adherence is crucial for the optimal control of diabetes [32]. For the item on smoking status, the available answers are “Yes” and “No”; For other domains, the domain score is computed as the average rating of the corresponding items, and the possible domain scores ranged from 0 to 7, with a higher score indicates better self-management behaviors. The reliability and validity of both the original and the Chinese versions of SDSCA have been well supported [5, 33]. In the current study, the Cronbach’s α coefficients for the subscales of general diet, physical activity, blood glucose monitoring and foot care were 0.87, 0.83, 0.91 and 0.76, respectively. However, the Cronbach’s α coefficient for the diabetes-specific diets subscale was 0.37, suggesting poor internal consistency reliability. This result coincides with the low inter-item correlation of the two items reported by the instrument developers [5]. Therefore, the two items were recommended to be analyzed separately.

HbA1c was measured with the standardized method of high-performance liquid chromatography by independent laboratories in the research sites.

Self-efficacy was measured with the Diabetes Management Self-Efficacy Scale (DMSES) [34]. DMSES has 20 items that assess participants’ confidence level in managing diabetes self-care activities regarding blood glucose control, foot care, medication, diet and physical activity. Responses are rated on an 11-point scale in which 0, 5 and 10 correspond to “Cannot (do) at all”, “Maybe yes/maybe no” and “Certainly can (do)”, respectively. Thus, the possible total score ranged from 0 to 200, with the higher scores indicating greater self-efficacy. The DMSES has been translated and evaluated for psychometric properties among Chinese type 2 diabetes population. The Chinese version of the DMSES demonstrated adequate reliability and validity [35]. In the current study, the Cronbach’s α coefficient of the overall scale was 0.94.

The measurement of subjective data was carried out by four independent research assistants who were blinded for the group allocation. A training session with competency assessment was arranged and ten individuals with type 2 diabetes were recruited for a pilot data collection. For each domain score of the SDSCA and the total score of the DMSES, the inter-rater agreement among the four outcome assessors was excellent with an Intra-Class Correlation or Cohen’s kappa coefficient of > 0.9.

### Procedure

Upon obtaining written informed consent, baseline data collection was conducted by trained research assistants while biomedical outcome indicators were measured by independent laboratories in the research sites. Sequentially, the participants were randomly allocated to receive either the nurse-led integrative medicine-based structured education program or the usual care. Post-intervention measurements were conducted immediately (T1) and 12 weeks (T2) following the completion of the 4-week interventions by the same research assistants or laboratories. All psychosocial and behavioral data were collected via face-to-face interviews, and the outcome assessors had no information about the group allocation.

### Ethical considerations

Ethical approval was obtained from the Joint Chinese University of Hong Kong-New Territories East Cluster Clinical Research Ethics Committee (reference number: 2016.676), and permissions were received from the involved hospitals. Written informed consents were obtained from the participants before the commencement of the study. The study was conducted with the participants’ rights and safety protected by adhering to local laws, Hong Kong Personal Data (Privacy) Ordinance, and the Declaration of Helsinki.

### Statistical analysis plan

Statistical analysis was performed using IBM SPPS, version 22. Appropriate descriptive statistics were used to summarize the characteristics of the participants. The independent-sample t-test, Mann-Whitney U test, Chi-square test, or Fisher’s exact test was employed, where appropriate, to detect any statistical difference in the social-demographic or clinical profiles, or outcome variables between the two study groups at baseline. Such comparisons were also conducted between the program completers and dropouts to assess potential attrition bias.

The generalized estimating equation (GEE) models were performed to assess the group- and time- differences, and the group-by-time interactions of each outcome variable across the time points. The GEE models consider the within-subject correlation of longitudinal data and allows for missing data (handled with the built-in quasi-likelihood estimation method) and time-varying covariate [36]. Thus, the intention-to-treat (ITT) principle was observed without the imputation of missing data. Effect size estimates were calculated for continuous variables with Cohen’s d and for categorical variables with phi (φ) at the 12th-week follow-up.

## Results

### Recruitment and attrition

**Fig. 1:**
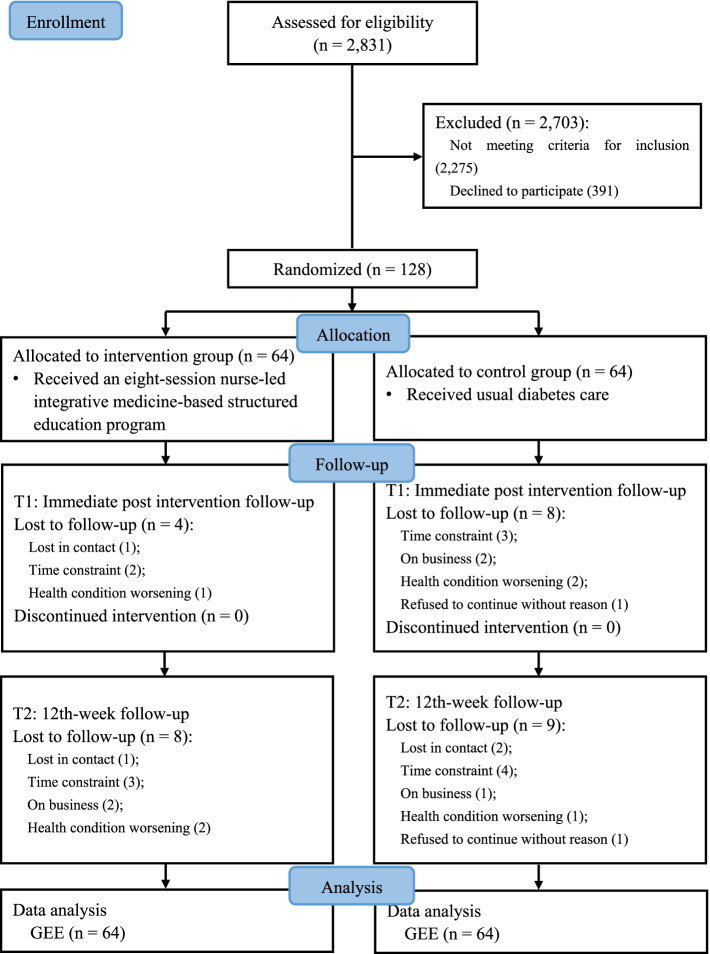
Flow diagram of participant recruitment, allocation, follow-up and data analysis

As shown in the CONSORT flow diagram, 128 eligible participants were sampled as planned (Fig.1). A total of 21 participants lost to follow-up at the immediate post-intervention and/or the 12th-week follow-up, with the major reason of lack of time or conflict of schedule. The comparison of baseline social-demographic and clinical characteristics between the completers and dropouts detected no significant difference. Of the 64 participants in the intervention group, 47 attended all of the eight educational sessions, representing an intervention adherence rate of 73.4%.

### Social-demographic and clinical characteristics of the participants

The average age of the participants was 57.43 years (standard deviation = 10.62, ranged from 33.7 to 74.8). The baseline diabetes duration of all participants ranged from three to 9 months, with an average duration of 6.70 (standard deviation = 2.25) months, while their baseline HbA1c level ranged from 4.40 to 13.30%, with a mean of 6.76% (standard deviation = 1.22%). A larger proportion of the research participants were male (58.6%), married (89.1%), with an educational level of secondary education or above (97.7%), retired (59.4%), and having a public insurance (99.2%). Most of the participants had concurrent chronic diseases (66.4%), among which hypertension is the most frequently reported condition, with a concurrent rate of 39.8%. There was no significant difference between the intervention and control groups in social-demographic and clinical characteristics. The baseline characteristics of the total samples and the comparison between groups are presented in Table 2.

**Table 2:** Social-demographic and clinical characteristics of the total samples and the comparison between groups at baseline (*N* = 128)

Characteristics	Total sample (N = 128) [$\overline{X}\pm S$ /Median (IQR)/n (%)]	Intervention group (*N* = 64) [$\overline{X}\pm S$ /Median (IQR)/n (%)]	Control group (N = 64) [$\overline{X}\pm S$ /Median (IQR)/n (%)]	*p* value
**Gender**	–	–	–	–
Male	75 (58.6%)	37 (57.8%)	38 (59.4%)	0.858^*^
Female	53 (41.4%)	27 (42.2%)	26 (40.6%)	
**Age (years)**	57.43 ± 10.62	58.04 ± 10.46	56.83 ± 10.82	0.520^#^
**BMI**	23.71 ± 3.03	23.59 ± 2.90	23.82 ± 3.16	0.658^#^
Underweight	4 (3.2%)	2 (3.2%)	2 (3.2%)	0.775^*^
Normal	66 (52.0%)	32 (50.8%)	34 (53.1%)	
Overweight	44 (34.6%)	24 (38.1%)	20 (31.3%)	
Obesity	13 (10.2%)	5 (7.9%)	8 (12.4%)	
**Marital status**	–	–	–	–
Married	114 (89.1%)	57 (89.1%)	57 (89.1%)	1.00^*^
Single/Divorced/Widowed	14 (10.9%)	7 (10.9%)	7 (10.9%)	
**Educational leve**l	–	–	–	–
Primary school or below	3 (2.3%)	1 (1.6%)	2 (3.1%)	0.697^*^
Secondary school	65 (50.8%)	33 (51.6%)	32 (50.0%)	
College	34 (26.6%)	15 (23.4%)	19 (29.7)	
Undergraduate or above	26 (20.3%)	15 (23.4%)	11 (17.2%)	
**Occupational status**	–	–	–	–
Employed	48 (37.5%)	22 (34.4%)	26 (40.6%)	0.465^*^
Unemployed/Retired	80 (62.5%)	42 (65.6%)	38 (59.4%)	
**Family average income (monthly in CNY)**	4250 (3000, 6000)	4000 (3000, 5250)	4500 (3000, 6000)	0.867^§^
**Insurance**	–	–	–	–
Public insurance	127 (99.2%)	63 (98.4%)	64 (100%)	1.000^¶^
Commercial insurance	1 (0.8%)	1 (1.6%)	0 (0.0%)	
**Diabetes family history**	–	–	–	–
Yes	40 (31.3%)	18 (28.1%)	22 (34.4%)	0.624^*^
No	71 (55.5%)	36 (56.3%)	35 (54.7%)	
Unclear	17 (13.2%)	10 (15.6%)	7 (10.9%)	
**Diabetes duration (months)**	6.70 ± 2.25	6.86 ± 2.21	6.53 ± 2.30	0.412^#^
**HbA1c (%)**	6.76 ± 1.22	6.66 ± 1.09	6.86 ± 1.34	0.368^#^
< 6.5%	54 (42.5%)	29 (46.0%)	25 (39.1%)	0.427^*^
≥ 6.5%	73 (57.5%)	34 (54.0%)	39 (60.9%)	
**Low blood glucose reaction**	–	–	–	–
Yes	35 (27.6%)	21 (33.3%)	14 (21.9%)	0.148^*^
No	92 (72.4%)	42 (66.7%)	50 (78.1%)	
**Treatment regimen**				
No medical treatment/Lifestyle modification	32 (25.6%)	15 (23.8%)	17 (27.4%)	0.941^*^
Oral medicine only	74 (59.2%)	39 (61.9%)	35 (56.5%)	
Insulin only	2 (1.6%)	1 (1.6%)	1 (1.6%)	
Oral medicine & insulin	17 (13.6%)	8 (12.7%)	9 (14.5%)	
**Acute complications**	–	–	–	–
Yes	12 (9.4%)	6 (9.4%)	6 (9.4%)	1.000^*^
No	116 (90.6%)	58 (90.6%)	58 (90.6%)	
**Chronic complications**	–	–	–	–
Yes	28 (21.9%)	18 (28.1%)	10 (15.6%)	0.087^*^
No	100 (78.1%)	46 (71.9%)	54 (84.4%)	
**Concurrent chronic diseases**	–	–	–	–
Yes	85 (66.4%)	43 (67.2%)	42 (65.6%)	0.852^*^
No	43 (33.6%)	21 (32.8%)	22 (34.4%)	

Note: $\overline{X}\pm S$: mean ± standard deviation; *IQR* Interquartile range, n (%): count (percentage); *p* value: the probability of a true null hypothesis; *BMI* Body mass index, *CNY* Chinese Yuan, *HbA1c* Glycated Hemoglobin A; ^*^: Chi-square tests; ^#^: independent-samples t tests; ^§^: Mann-Whitney U test; ^¶^: Fisher’s exact test

### Intervention effects on diabetes self-management behaviors

The baseline and post-test values in the SDSCA subscales are presented in Table 3. Participants in the intervention group exhibited significantly better self-management behaviors in specific diet regarding intake of fruits and vegetables at both the immediate post-intervention and the 12th-week follow-ups (β = 1.02, *p* = 0.011 and β = 0.98, *p* = 0.016, respectively), specific diet regarding intake of high-fat foods at the immediate post-intervention follow-up (β = 0.83, *p* = 0.023), blood glucose monitoring at the 12th-week follow-up (β = 0.64, *p* = 0.004), foot care at both the immediate post-intervention and 12th-week follow-ups (β = 1.80, *p* < 0.001 and β = 2.02, *p* < 0.001, respectively), and medication management at both the immediate post-intervention and 12th-week follow-ups (β = 0.83, *p* = 0.005 and β = 0.95, *p* = 0.003, respectively), as demonstrated by the results of GEE models (Table 4).

**Table 3:** Comparison of outcome variables between groups and effect size of the intervention over the study period (N = 128)

Variables	Control group* (N = 64)	Intervention group* (N = 64)	Between-group *p* value	Effect size (Cohen’s d/φ)
**HbA1c (%)**				
Baseline	6.86 ± 1.34	6.66 ± 1.09	0.368	–
T2 follow-up	6.97 ± 1.18	6.57 ± 0.84	0.041	0.39
**Diabetes self-management behaviors (SDSCA)**				
General diet				
Baseline	5.04 ± 2.07	5.36 ± 1.80	0.361	–
T1 follow-up	5.44 ± 1.16	5.78 ± 1.10	0.102	–
T2 follow-up	5.30 ± 1.17	5.88 ± 0.94	0.005	0.55
Specific diet regarding intake of fruits and vegetables				
Baseline	4.28 ± 2.60	4.30 ± 2.54	0.965	–
T1 follow-up	4.13 ± 2.23	5.12 ± 1.69	0.008	–
T2 follow-up	4.08 ± 2.07	5.07 ± 1.67	0.007	0.53
Specific diet regarding intake of high fat foods				
Baseline	3.89 ± 2.29	4.00 ± 2.46	0.797	–
T1 follow-up	4.00 ± 1.53	4.90 ± 1.81	0.005	–
T2 follow-up	4.10 ± 1.33	4.62 ± 1.56	0.066	0.36
Physical activity				
Baseline	5.02 ± 2.21	4.85 ± 2.18	0.673	–
T1 follow-up	5.17 ± 1.88	5.10 ± 1.93	0.826	–
T2 follow-up	4.92 ± 1.76	5.04 ± 1.73	0.721	0.07
Blood glucose monitoring				
Baseline	3.10 ± 2.18	2.91 ± 2.33	0.643	–
T1 follow-up	3.09 ± 2.08	3.14 ± 2.19	0.895	–
T2 follow-up	2.89 ± 1.82	3.30 ± 2.08	0.273	0.21
Foot care				
Baseline	3.59 ± 2.66	3.42 ± 2.74	0.719	–
T1 follow-up	3.46 ± 2.35	5.25 ± 1.99	< 0.001	–
T2 follow-up	3.47 ± 2.27	5.46 ± 1.69	< 0.001	0.99
Smoking status (non-smoker to smoker ratio)				
Baseline	43:21	46:18	0.565	
T1 follow-up	39:17	47:13	0.285	
T2 follow-up	40:14	49:8	0.116	0.149
Medication management				
Baseline	4.84 ± 3.01	5.44 ± 2.71	0.248	–
T1 follow-up	4.68 ± 3.04	6.22 ± 1.78	0.001	–
T2 follow-up	4.61 ± 3.02	6.18 ± 1.85	0.002	0.63
**Self-efficacy (DMSES)**				
Baseline	153.32 ± 37.62	147.90 ± 36.53	0.420	–
T1 follow-up	154.44 ± 31.99	157.29 ± 30.16	0.625	–
T2 follow-up	150.17 ± 32.75	155.52 ± 28.95	0.368	0.17

Note: *p* value: the probability of a true null hypothesis; Cohen’s d: effect size for continuous variables; φ: phi, effect size for categorical variables; *HbA1c* Glycated Hemoglobin A, *SDSCA* Summary of Diabetes Self-Care Activities, *DMSES* Diabetes Management Self-Efficacy Scale. *: in mean ± standard deviation unless specified

**Table 4:** GEE models for the comparison of outcome variables over the study period (N = 128)

Variables	Group-difference		Time1-difference		Time2-difference		Group*Time1-difference		Group*Time2-difference	
	β (95% CI)	*p* value	β (95% CI)	*p* value	β (95% CI)	*p* value	β (95% CI)	*p* value	β (95% CI)	*p* value
**HbA1c (%)**	−0.20 (− 0.62, 0.23)	0.365	–	–	**0.18** **(0.05, 0.30)**	**0.006**	–	–	**−0.32** **(− 0.49, − 0.14)**	**< 0.001**
**Diabetes self-management behaviors (SDSCA)**										
General diet	0.30 (−0.38, 0.98)	0.383	0.37 (−0.04, 0.77)	0.075	0.31 (−0.08, 0.70)	0.114	0.01 (−0.49, 0.51)	0.967	0.19 (−0.32, 0.70)	0.459
Specific diet regarding intake of fruits and vegetables	−0.04 (− 0.94, 0.86)	0.934	−0.16 (− 0.62, 0.29)	0.483	−0.12 (− 0.59, 0.36)	0.635	**1.02** **(0.24, 1.80)**	**0.011**	**0.98** **(0.18, 1.78)**	**0.016**
Specific diet regarding intake of high fat foods	0.07 (−0.75, 0.88)	0.875	−0.01 (− 0.48, 0.45)	0.958	0.09 (−0.38, 0.55)	0.717	**0.83** **(0.12, 1.55)**	**0.023**	0.50 (−0.20, 1.20)	0.163
Physical activity	−0.15 (− 0.91, 0.62)	0.702	0.10 (−0.27, 0.48)	0.586	−0.15 (− 0.56, 0.26)	0.473	0.15 (−0.31, 0.62)	0.525	0.39 (−0.12, 0.90)	0.132
Blood glucose monitoring	−0.25 (−1.02, 0.53)	0.532	−0.03 (− 0.24, 0.17)	0.751	−0.20 (− 0.51, 0.12)	0.218	0.36 (−0.01, 0.72)	0.057	**0.64** **(0.20, 1.08)**	**0.004**
Foot care	−0.17 (−1.1, 0.76)	0.717	−0.04 (− 0.46, 0.39)	0.863	−0.01 (− 0.46, 0.44)	0.962	**1.80** **(1.05, 2.54)**	**< 0.001**	**2.02** **(1.27, 2.77)**	**< 0.001**
Smoking status (non-smoker to smoker ratio)	1.23 (0.58, 2.62)	0.593	1.13 (0.85, 1.51)	0.392	1.32 (0.91, 1.91)	0.141	1.38 (0.85, 2.25)	0.195	1.95 (0.95, 4.00)	0.067
Medication management	0.56 (−0.43, 1.55)	0.267	− 0.08 (− 0.27, 0.12)	0.441	− 0.13 (− 0.33, 0.08)	0.230	**0.83** **(0.25, 1.41)**	**0.005**	**0.95** **(0.31, 1.58)**	**0.003**
**Self-efficacy (DMSES)**	−3.60 (−15.71, 8.50)	0.560	**−2.90** **(−5.73, −0.08)**	**0.044**	**−3.43** **(−6.47, −0.39)**	**0.027**	**8.73** **(3.34, 14.12)**	**0.001**	**9.71** **(4.03, 15.38)**	**0.001**

Note: β: difference in values; *p* value: the probability of a true null hypothesis; *HbA1c* Glycated Hemoglobin A, *SDSCA* Summary of Diabetes Self-Care Activities *DMSES* Diabetes Management Self-Efficacy Scale; Group-difference: Difference in indicator values between groups at baseline; Time-difference: Within group change in indicator values in the control group; Group*Time-difference: Additional change of the intervention group in indicator values compared to the control group; *CI* Confidential interval

### Intervention effects on HbA1c and self-efficacy

The baseline and post-test values in HbA1c and DMSES are presented in Table 3. Participants in the intervention group demonstrated a significant decrease of 0.32% (*p* < 0.001) in HbA1c compared to those in the control group (Table 4). The intervention also introduced significant improvements in self-efficacy at both the immediate post-intervention and the 12th-week follow-ups (β = 8.73, *p* < 0.001 and β = 9.71, *p* < 0.001, respectively).

No adverse event associated with the intervention was reported during the study period.

## Discussion

This study conceptualized an evidence-based, culturally appropriate, nurse-led integrative medicine-based structured education program and evaluated its effectiveness among individuals with newly diagnosed type 2 diabetes employing a multi-center randomized controlled trial. The results demonstrated beneficial effects on diabetes self-management behaviors, glycemic control and self-efficacy.

### Intervention effects on diabetes self-management behaviors

Self-management is crucial for individuals with diabetes. Adequate diabetes self-management has been consistently found to be associated with optimal glycemic control and the overall well-being of individuals with diabetes [37]. The international and national institutions, including NICE, ADA, ADCES, and China Diabetes Society, recommend providing prompt diabetes self-management education and training to individuals with the condition. Diabetes self-management education/training aims at helping individuals with the condition to maintain, mainly by their own efforts, the best possible diabetic control and overall health.

Both dietary patterns and behaviors can significantly influence the control over glycemic level of individuals with diabetes [38]. In the current study, participants in the intervention group were instructed to make individualized dietary plans according to their individual characteristics, for example, their body weight and activity level. They were also educated about how to identify healthy dietary behaviors, such as improving the proportion of vegetables and fruits in meals, preparing less fried foods and having regular meals, and unhealthy dietary behaviors, for example, overconsumption of salt and high-fat foods. Various strategies evolved from the Health Belief Model and Self-Efficacy Theory were employed to facilitate the development of healthy dietary behaviors. Out of expectation, however, this study identified no effect of the intervention on general diet self-management behaviors. As none of the seven eligible trials included in the systematic review regarding the effectiveness of structured diabetes education provided independent data on self-management behaviors regarding general diet [13], we extended the comparison of our findings to existed theory-based diabetes education programs. The results of this study contradicted the findings of some previous trials where beneficial effects were frequently reported [39]. The absence of the hypothesized between-group difference in the current study could be attributed to a ceiling effect as a satisfactory baseline level (mean = 5.20) was reported by the participants, leaving limited room for further improvement.

Significant improvements in diabetes-specific diet management behaviors were observed in this study. As existed trials frequently pooled the two items for data analysis, comparing the findings on such self-management behaviors was not feasible. Researchers are suggested to use the outcome measurement instruments in the standard approach. In the intervention session on healthy eating, participants in the intervention group were encouraged to increase the proportion of vegetables in their meals and were instructed on food exchange so that they would be able to replace some of the high-carbohydrate foods with vegetables. A longstanding misleading concept among many Chinese people is that individuals with diabetes should avoid fruits because they are sweet and thus can significantly elevate the blood glucose level [40]. However, the effect of fresh fruits on elevating blood glucose level is far less influential than an equivalent amount of noodles or rice, which are rich in carbohydrates [41]. Differences between sugars, carbohydrates and fibers, and how they influence blood glucose were clarified so that the participants could gain a correct concept on fruit intake. Avoiding overconsumption of high-fat foods was also addressed in the program. Thus, it is rational to anticipate such improved performance. However, the improvement in diabetes-specific diet management regarding intake of high-fat foods did not sustain at the 12-th week follow-up. More intensive interventions with reinforcement strategies may produce a better result. A reflection session following each educational session and the use of mobile phone applications to provide boosting sessions could be effective alternatives to improve the intervention intensity and to reinforce the intervention effects [42].

No beneficial effect of the intervention on physical activity was detected, which was inconsistent with the findings of earlier structured education programs [14, 15]. The non-significant finding of the current study could be attributed to the ceiling effect, as the participants had a comparably high level of physical activity at baseline (mean = 4.93). It should be noted that the measured level of self-management behaviors regarding regular physical activity could be invalid due to the nature of the instrument used. In one of the two items measuring self-management behaviors regarding physical activity, the participants were asked about the number of days they did exercise for at least 30 minutes in the past week. The investigated exercise includes walking and doing household work, which could be too low in intensity and thus would not be effective for individuals with diabetes [2, 43]. Consequently, the potential beneficial effect of the intervention could be masked as, in this situation, the measured post-intervention physical activity behaviors in the control group could include a larger proportion of ineffective physical activities compared to those in the intervention group. Using wearable devices to accurately measure physical activity is recommended in further studies.

The current study showed favorable effects on promoting self-management behaviors regarding blood-glucose monitoring at both the immediate post-intervention and the 12th-week follow-ups, even though the immediate effect was just about to reach the significant level. This finding is consistent with a previous study of structured education [15], however, contradicted the result of another structured education program [20]. Low power could be the reason for the non-significant result in Vincent’s study as only 20 participants were recruited [20]. The gradually improved performance in blood glucose testing concurred with the Self-Efficacy Theory as the successful performance of self-monitoring of blood glucose would improve self-efficacy in this aspect serving as a source of performance accomplishments [27, 28], which, in return, could contribute to the further improvements of the behavior.

Consistent with the findings of an earlier structured education program [15], this study showed a significant effect in promoting foot care self-management behaviors at both the immediate post-intervention and the 12th-week follow-ups. However, a contradictory result was reported in another structured education program [20]. The non-significant findings reported by Vincent could be attributed to its low power and the satisfactory level of foot care behaviors of the research participants at baseline [20]. The effect of the program on promoting foot care self-management behaviors in the current study is quite large. Such a large effect could be explained by the poor performance of the participants at baseline. Cost-efficiency could be another factor that contributed to the large effect, as the daily foot care behaviors could be conducted at no cost.

The effect of the intervention on self-management in smoking was non-significant. The finding of the current study was consistent with the result of a previous structured education program, which suggested that there was no significant difference in the smoking status between the compared groups at the 4th-month follow-up [14]. Hoverer, the investigators observed significant beneficial effects at the 8th-month and 12th-month follow-ups. Such non-significant short-term findings in the previous study and the current study could be explained by the fact that successful smoking cessation is a common challenge among various populations [44]. More intensive smoking cessation interventions could be considered. Considering the promising longer-term findings in the earlier trial and the tendency in the increasing non-smoker-to-smoker ratio in the current study, the long-term effectiveness of structured education programs on smoking self-management behaviors could be expected.

This study showed a significant result that favored the effectiveness of the intervention in improving self-management behaviors regarding medication management. This finding of the current study contradicted the result of a previous study of structured education with low power [20]. Discrepancies were found when we extended the comparison to a broader spectrum of literature by including theory-based diabetes education programs [39]. Even though the self-management level of medication among Chinese individuals with diabetes is at a relatively high level, as is found in both the current study and relevant studies [45], the individuals frequently tried to self-adjust the dosage and frequency of their medications either because they think their condition improved or because they hope to reduce related costs [46]. In the current study, participants in the intervention group were instructed on detailed medication management strategies, such as regular medication taking, surveillance of adverse drug effects and scheduled medication adjustment planned with the healthcare professionals. This could help explain the further improvement in self-management of medication in the intervention group despite the relatively high-level of medication self-management behaviors at baseline.

### Intervention effects on glycemic control and self-efficacy

#### Glycemic control

HbA1c reflects the average glycemic level over an approximate interval of three months and has a strong predictive value for diabetes complications, and thus is regarded as the golden indicator for the assessment of glycemic control. In general, a reasonable HbA1c target for non-pregnant adults with diabetes is suggested to be < 7% (53 mmol/mol). It is suggested that a reduction of ≥0.5% in HbA1c could be considered as clinically sound [47]. The result of this study showed a significantly greater reduction in the level of HbA1c in the intervention group compared to the control group at the 12th-week follow-up, favoring the effectiveness of the intervention program in improving glycemic control. The finding regarding the effectiveness of structured education in lowering HbA1c level was consistent with the results of several previous studies of structured education [15, 16, 18]. Meanwhile, an equivalent number of previous structured education programs demonstrated contradicting results [14, 19–21]. However, the insignificant findings of these studies could be attributed to a low power [20], a ceiling effect (the participants had relatively good glycemic control at baseline) [20, 21, 48], the utilization of an active comparator [14, 17], and an inappropriate time point for intervention initiation (immediate structured education for individuals with newly diagnosed diabetes in the course of intensive medical treatments) [14, 17] or model of intervention delivery (self-instructed without supervision) [19]. In the development of this program, the methodological flaws were adequately addressed to guarantee its soundness. The result of a beneficial effect on HbA1c level was also consistent with the findings of a systematic review with meta-analysis, in which the reviewers concluded that group-based diabetes education can significantly lower HbA1c level by a mean difference of 0.44 to 0.87, depending on the length of follow-up [25].

Despite the significant findings regarding the effectiveness of this program in lowering HbA1c level, it should be noted that the observed intervention effect was not large enough to reach a clinically significant level [47]. A ceiling effect could be a possible explanation for the relatively small effect size on lowering HbA1c level as the average baseline HbA1c level of the participants was quite low (6.76%) [48]. Nevertheless, a tendency of increase in HbA1c level was observed in the control group, whereas the intervention group demonstrated a tendency of decrease. Such deviating tendencies between groups could be meaningful considering a suboptimal glycemic control has been frequently demonstrated to be associated with long diabetes duration, if not intervened [49].

#### Self-efficacy

Self-efficacy decides individuals’ intention and compliance to health-promoting behaviors, and further influences the glycemic control of individuals with diabetes. Research evidence has frequently suggested an association between self-efficacy and glycemic control [49]. In this study, various strategies were employed to enhance the level of self-efficacy of the participants in the intervention group. The result of this study showed a significant benefit of the intervention program in improving the level of self-efficacy compared to the usual care at both the immediate post-intervention (β = 8.73, *p* = 0.001) and the 12th-week (β = 9.71, *p* = 0.001) follow-ups. The findings of this study were consistent with the results of existed studies of structured education [15, 19, 21] except the one under the criticism of being of low power. One of the common features of the effective structured education programs, including the current study, is the utilization of the self-efficacy/empowerment theory to inform the development of the programs. In view of the significant decrease in the level of self-efficacy in the control group and the significant increase in the intervention group in the current study, and the association between low level of self-efficacy and suboptimal glycemic control, such beneficial effect of the intervention program could be of great significance in the long run.

### Strengths and limitations

This study has several remarkable strengths. Firstly, it is among the few eligible structured education programs for individuals with diabetes. The incorporation of traditional Chinese medicine-based lifestyle interventions and the demonstrated effectiveness provided an option of culture-tailored structured education for Chinese individuals with type 2 diabetes. Secondly, the study was conducted in four hospitals, which enhanced the representativeness of the research participants. Thirdly, data were analyzed adhering the ITT principle, so that the confounding effects of covariates arising post randomization could be minimized.

Despite its important contribution to the knowledge body, this study has some limitations which should be taken into consideration when interpreting the findings. Even though multiple research sites were involved, the hospitals were all university-affiliated tertiary hospitals. Due to the heterogeneity in the resources and quality of service provided in different levels of hospitals in China, the representativeness of the research participants could still be limited. A series of high-level criteria were utilized to select the intervention deliverers in this study. Admit that competent intervention deliverers could facilitate the smooth progression of a program, such competent personnel may not always be available for diabetes education activities under the real-world situation considering the heavy clinical workload and the educational level of the nursing professionals in China. As the skills and competence of the intervention deliverers may be the only “active ingredient” that leads to the observed results [50], the generalizability of the program could be compromised. Besides, this study failed to record the participants’ actual compliance to the recommended health-promoting strategies. Instead, diabetes self-management behaviors were measured in a subjective approach using a scale, which could produce biased results and thus lead to inaccurate statistical inferences. What is more, this study only examined the immediate and short-term effectiveness of the program, remaining the longer-term effectiveness of the program unknown.

### Implications

#### Implications for further research

To facilitate a comprehensive comparison of the intervention effects, researchers are recommended to measure different domains of diabetes self-management behaviors independently. Meanwhile, more objective and reliable instruments should be considered when collecting behavioral outcomes. The investigation on the long-term effectiveness of this culture-tailored structured education program in various settings (such as primary healthcare service centers, rehabilitation centers and the community) and its cost-effectiveness are guaranteed in order to determine its generalizability. Further qualitative researches are worthwhile to explore the intervention mechanism and client experience.

#### Implications for nursing practice

The significant findings regarding the effectiveness of this program on patient-centered outcomes support the commencement of structured education programs for individuals with newly diagnosed type 2 diabetes. Structured diabetes education should cover all the important aspects of diabetes self-management, and be delivered in an interactive manner that facilitates timely communication, highlights individualization, and promotes the proactivity of the participants. Group-based education should be considered as the prior format as it is equivalently effective but more cost-efficient when compared with individual diabetes education [25]. Considering a decrease in the intervention effect at the 12-th follow-up compared to the immediate post-intervention follow-up, regular reinforcement sessions should be provided to maintain the beneficial effects of educational programs in the long run. Such reinforcement sessions could be delivered in various convenient ways that benefited from the advances of technology, such as phone calls, short messages and mobile applications.

## Conclusions

This study developed a nurse-led integrative medicine-based structured education program based on a theoretical framework of the Health Belief Model and Self-Efficacy Theory, most updated diabetes management guidelines and relevant systematic reviews for individuals with newly diagnosed type 2 diabetes. The program exhibited beneficial effects on multiple diabetes self-management behaviors, glycemic control and self-efficacy. Further investigations on the long-term effectiveness and the generalizability of the program to a broader range of populations are worthwhile.

## Supplementary Information

**Additional file 1.**

## Data Availability

The datasets generated and analyzed during the current study are not publicly available due to privacy protection and ethical considerations but are available from the corresponding author on reasonable request.

## Abbreviations

ADA: American Diabetes Association
ADCES: Association of Diabetes Care & Education Specialists
CONSORT: Consolidated Standards of Reporting Trials
DESMOND: Diabetes Education and Self-Management for Ongoing and Newly Diagnosed
DMSES: Diabetes Management Self-Efficacy Scale
DSMES: Diabetes self-management education and support
GEE: Generalized estimating equation
HbA1c: Glycated Hemoglobin A
NICE: National Institute for Health and Care Excellence
SDSCA: Summary of Diabetes Self-Care Activities
SD: Standard deviation
TCM: Traditional Chinese medicine
