# Supplementary figures and images for: The effects of a nurse-led integrative medicine-based structured education program on self-management behaviors among individuals with newly diagnosed type 2 diabetes: a randomized controlled trial
Source: BMC Nurs. 2022 Aug 5;21:217. doi: 10.1186/s12912-022-00970-7 (PMC9354282; doi:10.1186/s12912-022-00970-7)

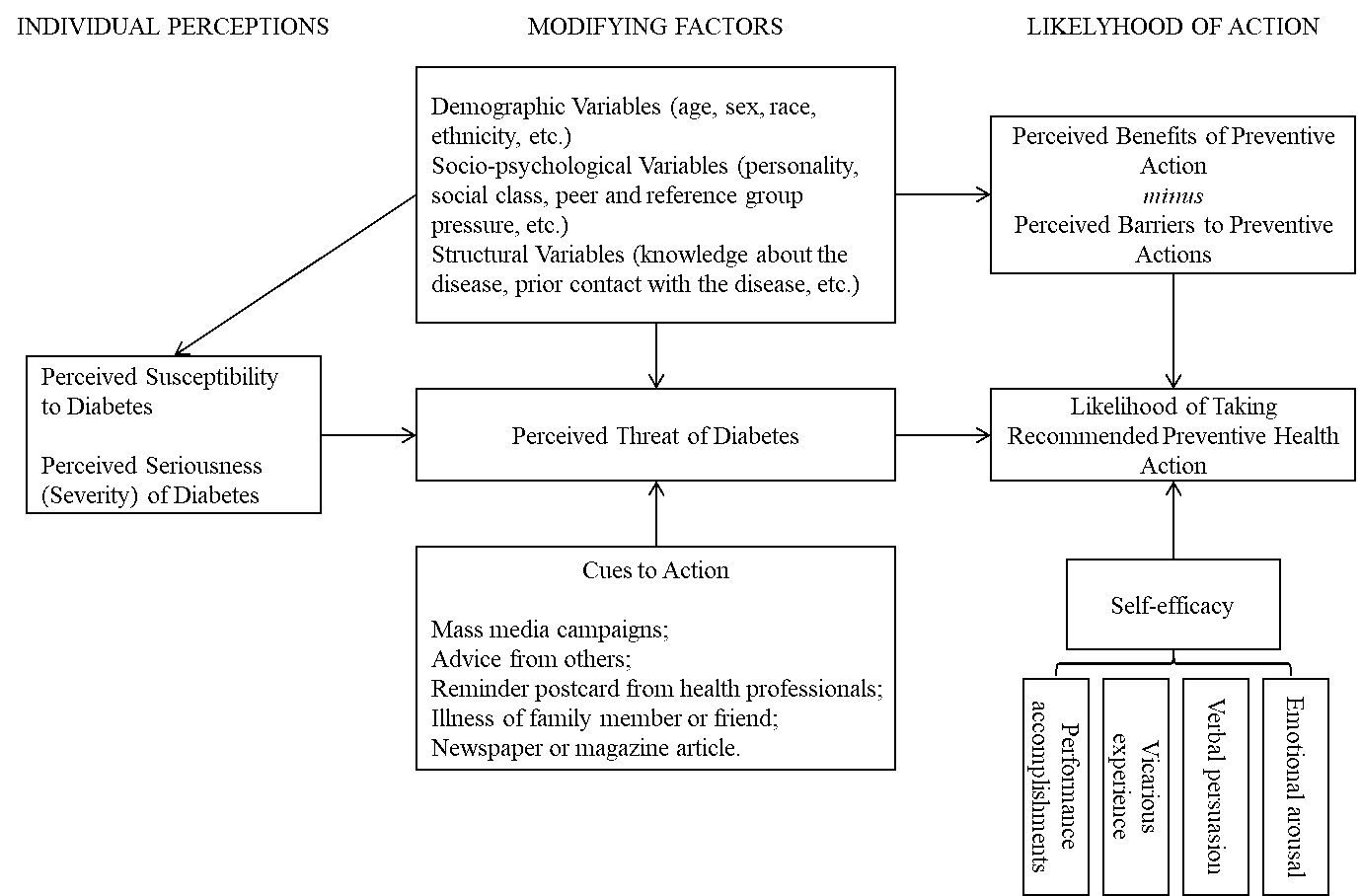


Supplementary Fig.1. Theoretical framework of the Health Belief Model and Self-Efficacy Theory

Supplement: Supplementary file 1 — Additional file 1. [file 12912_2022_970_MOESM1_ESM.docx]
